# Supplementary material for: Electrical Stimulation Increases Axonal Growth from Dorsal Root Ganglia Co-Cultured with Schwann Cells in Highly Aligned PLA-PPy-Au Microfiber Substrates
Source: Int J Mol Sci. 2022 Jun 7;23(12):6362. doi: 10.3390/ijms23126362 (PMC9223746; doi:10.3390/ijms23126362)
Supplement: Supplementary file 1 [file ijms-23-06362-s001.zip › ijms-1734477-supplementary.pdf]

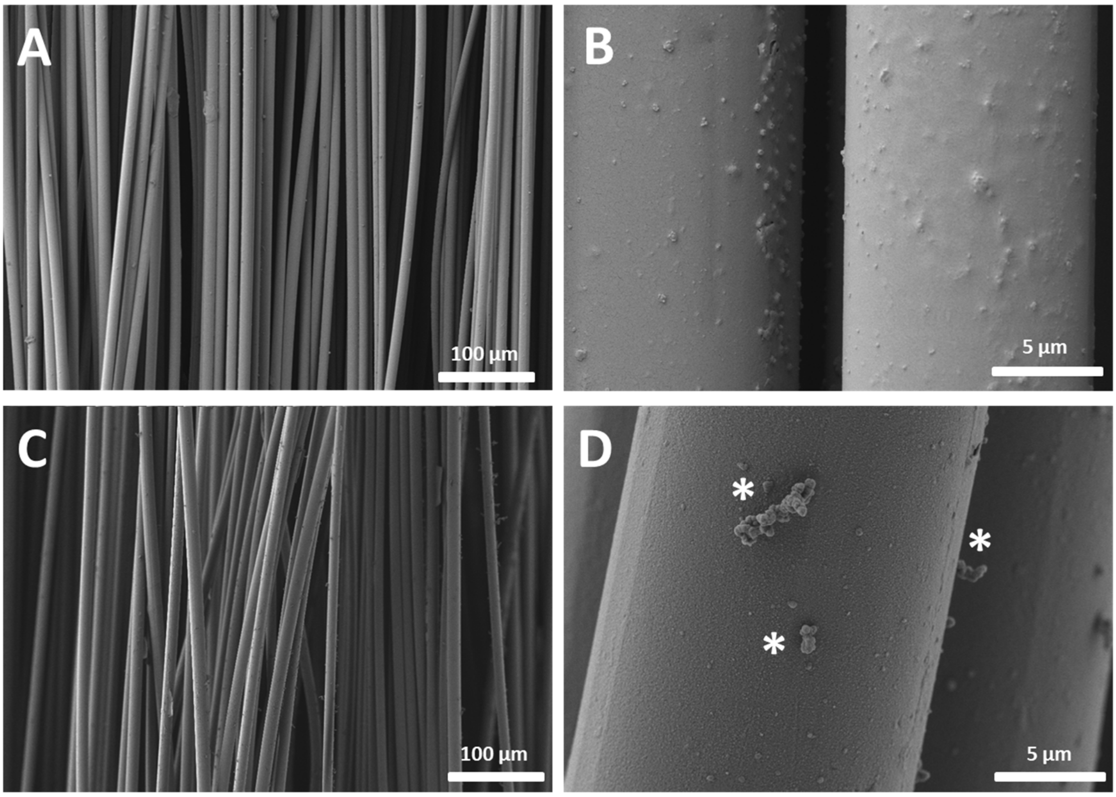

Figure S1. **A**: PLA microfiber lane previously to the PPy coating. Microfibers with a diameter of 10 µm were employed. **B**: Detail of the PLA microfibers previously to the PPy coating. **C**: PLA-PPy microfiber lane. **D**: Detail of the PLA-PPy microfibers. A homogeneous PPy coating can be observed, with a fine grain texture. Only some aggregates (marked with *) were present and most of them disappeared with repeated washing.

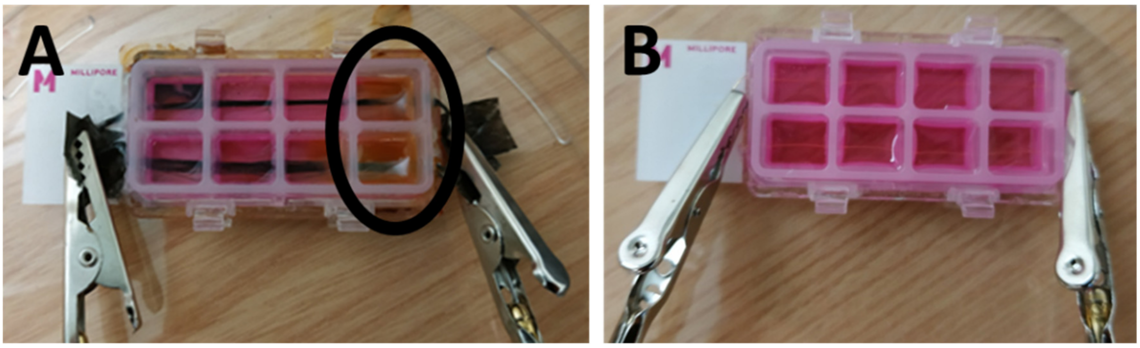

Figure S2. PLA-PPy microfibers (**A**) and gold microfibers (**B**) stimulated for 8 hours with direct current (DC). As can be observed, when PLA-PPy microfibers were employed, there was a change in the culture medium colour, that changed from pink to yellow. However, when gold microfibers were used, this effect did not appear.

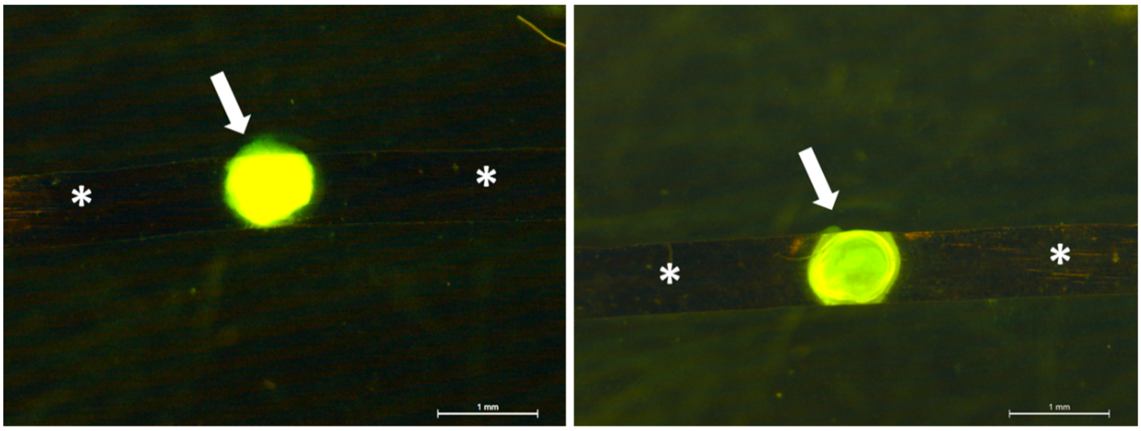

Figure S3. Chicken DRG (marked with arrows) seeded on top of a gold substrate (marked with *) after 4 days of cell culture. Immunostaining with β III Tubulin is observed in green colour. As can be observed, there is no axonal extension from the DRG bodies, indicating a poor adhesion of DRG bodies to the gold substrate.
